# Luteinizing hormone elevation in ovarian granulosa cell tumor: a case report and review of the literature

**DOI:** 10.1186/s13048-017-0327-2

**Published:** 2017-04-24

**Authors:** Shengyuan Ran, Qi Yu, Shan Deng, Ling Xu

**Affiliations:** Chinese Academy of Medical Sciences and Peking Union Medical College Hospital, Beijing, People’s Republic of China

**Keywords:** LH elevation, GCTs, Amenorrhea

## Abstract

**Background:**

Ovarian granulosa cell tumors (GCTs) are the most common type of potentially malignant ovarian sex cord-stromal tumor. GCTs often produce estrogen and/or progesterone; consequently, symptoms related to hyperestrogenism are common at diagnosis. Nonspecific symptoms or signs associated with this neoplasm include amenorrhea and changes in various sex hormone levels, which can be hard to diagnose or explain. The aims of this report were to describe a case of GCT with rare presentations and to review the pertinent literature.

**Case presentation:**

A 33-year-old woman presented with secondary amenorrhea and elevated LH with normal FSH and low estradiol levels. Laparoscopy revealed an ovarian GCT, and the patient underwent left adnexectomy and platinum-based chemotherapy. Removal of the ovarian GCT resulted in the normalization of LH levels and a return to spontaneous menses.

**Conclusions:**

The mechanisms responsible for elevations in serum LH due to GCTs require further investigation. However, addressing the patient’s clinical problems remains the most important treatment consideration.

## Background

GCTs are the most common type of potentially malignant ovarian sex cord-stromal tumor and comprise to 5% of all ovarian malignancies [1]. GCTs are functional tumors derived from the mesenchymal stroma of the gonad and typically presents with the elevation of sex steroids, primarily hyperoestrogenism [2]. Occasionally, other hormone changes may also be observed. Here, we report a case of histologically confirmed GCT that presented with secondary amenorrhea and elevation of only LH levels, with normal FSH and low estradiol levels and review the pertinent literature.

## Case presentation

A 33-year-old Chinese woman with a 2-year history of secondary amenorrhea was referred to the reproductive endocrinology clinic at Peking Union Medical College Hospital. The patient had experienced menarche at age 12, and her menstrual cycle had been regular until 5 years ago. At that time, the patient’s cycle length became irregular, with a range of 2 to 3 months. The patient did not present to the reproductive endocrinologist until her menstrual bleeding had completely stopped for 5 months.

The patient complained of hot flushes, night sweats and a 10-kg weight gain over the last 2 years (body mass index from 17 to 20.5 kg/m2). She had no clinical manifestations of hirsutism or acne. The patient’s breasts and pubic hair were well- developed (Tanner stage 5) with no galactorrhea. Abdominal examination failed to reveal any palpable masses. No family history of autoimmune disorders was reported.

Serum hormone assays revealed a negative serum human chorionic gonadotropin (hCG) and a high LH level of 30.12 IU/L. The other hormone levels were as follows: FSH, 7.49 IU/L; estradiol, 18.2 pg/ml; progesterone, 0.64 ng/ml; testosterone, 0.56 ng/dl; and prolactin, 7.12 ng/ml, with normal thyroid function. Routine laboratory tests and tumor markers were all within normal limits.

Pelvic ultrasonography examination demonstrated a 2.3 × 2.0-cm partial solid mass surrounded with a blood flow signal in the left ovary. The uterus was normal in size with an endometrial thickness of 0.3 cm. The right ovary was unremarkable. No free fluid or other pelvic masses were observed. Magnetic resonance imaging (MRI) revealed a 5.9 × 5.6-mm hypoenhancing mass in the pituitary gland.

The patient had received many treatment modalities during the 2-year period at other hospitals. LH levels on the second day of oral contraceptive (ethinyl estradiol and cyproterone acetate tablets) withdraw bleeding fell to 8.51 IU/L. The patient then presented to our department for further examination. Withdrawal bleeding could occasionally be induced by dydrogesterone (10 mg per day for 10 days per month), but this effect was inconsistent. The LH levels detected during this period fluctuated between 30 and 47 IU/L. Because she failed to maintain a normal menstrual cycle in the absence of medication (she only had two episodes of spontaneous menstruation during the entire treatment period), the patient was prescribed the sequential use of estrogen and progestin to maintain regular menstruation. The high LH level (34–42 IU/L) persisted throughout the treatment course. The patient was again advised to take oral contraceptives to observe the change in LH levels. The patient developed menstrual bleeding after taking pills, and the LH level on the second day of her menstruation decreased to 7.51 IU/L.

**Fig. 1:**
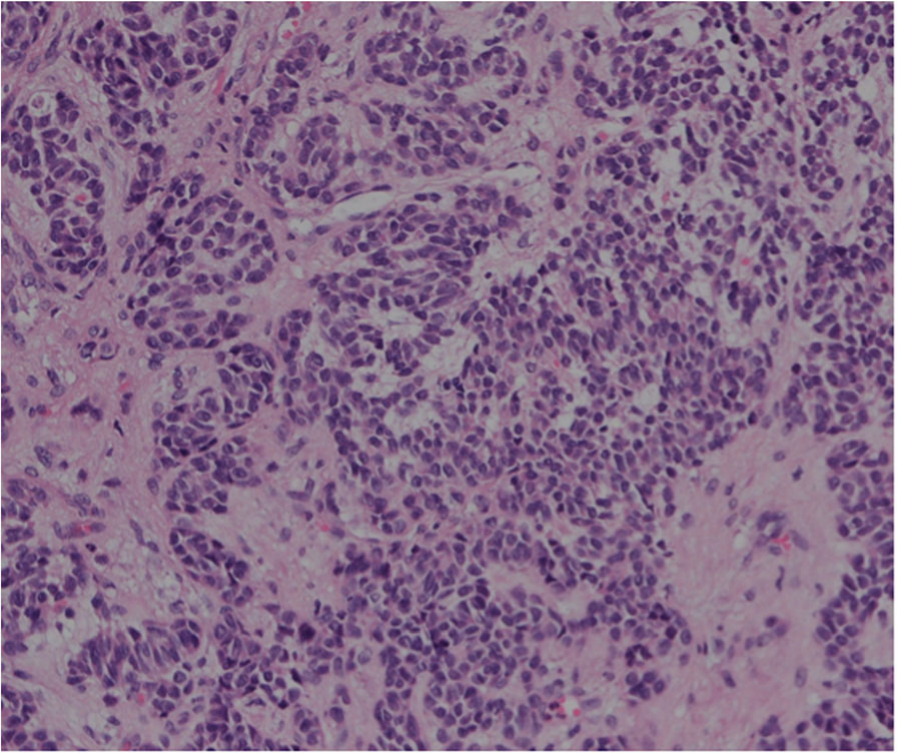
Histological ovarian tumor findings. The tumor exhibited typical features of a granulosa cell tumor. H&E, ×200

In general, the pattern of elevated LH (15.01–47.85 IU/L) persisted through the 2- year period with the exception of the period of oral contraceptive use. Repeated ultrasonography examinations during her treatment period revealed that the mass had gradually grown to a solid-appearing structure measuring 2.7 × 2.9 × 2.0 cm in size with a rich blood flow signal. An exploratory laparoscopy was performed. The solid ovarian tumor exhibited a yellow lipid-like appearance and was 3 cm in diameter with rich blood flow. There was no evidence of extra-ovarian disease. A rapid frozen pathology examination of the resected tumor indicated a sex cord-stromal tumor with low malignant potential. Because the parents of this patient refused to consent to a left salpingo-oophorectomy, left cystectomy was performed. The tumor was diagnosed as an adult-type GCT with active growth on the final pathological examination, with 10 to 12 mitotic figures per 10 high-power fields (Fig. 1). The cells stained immunopositive for inhibin A, Melan-A and CD99 but were immunonegative for LH. Two weeks after the surgery, the hormone levels were as follows: LH, 13.65 IU/L, and FSH, 4.9 IU/L.

Unilateral adnexectomy with preservation of the contralateral ovary and the uterus is now considered adequate surgical treatment for patients with GCTs. Younger patient age and early-stage of the disease are the most important predictors of improved disease-specific survival in GCTs [3]. Thus, the patient was strongly recommended to undergo left adnexectomy. Histological examination after the second surgery confirmed residual GCT tissue in the left ovary, and the tumor was diagnosed as GCT stage Ic [4]. Stage Ia GCT disease has an excellent prognosis after surgery and does not require adjuvant therapy [5]. Some authors suggest adjuvant therapy for stage Ic patients with a high mitotic index [3]. Thus platinum-based chemotherapy was applied in this case. Furthermore, reexamination of the pituitary gland by MRI after surgery revealed a 5.7 × 6.0-cm tumor that did not differ in size from previous measurements. Neurosurgery consultation suggested that the pituitary adenoma was clinically nonfunctional and suggested yearly MRI for 2 years.

**Fig. 2:**
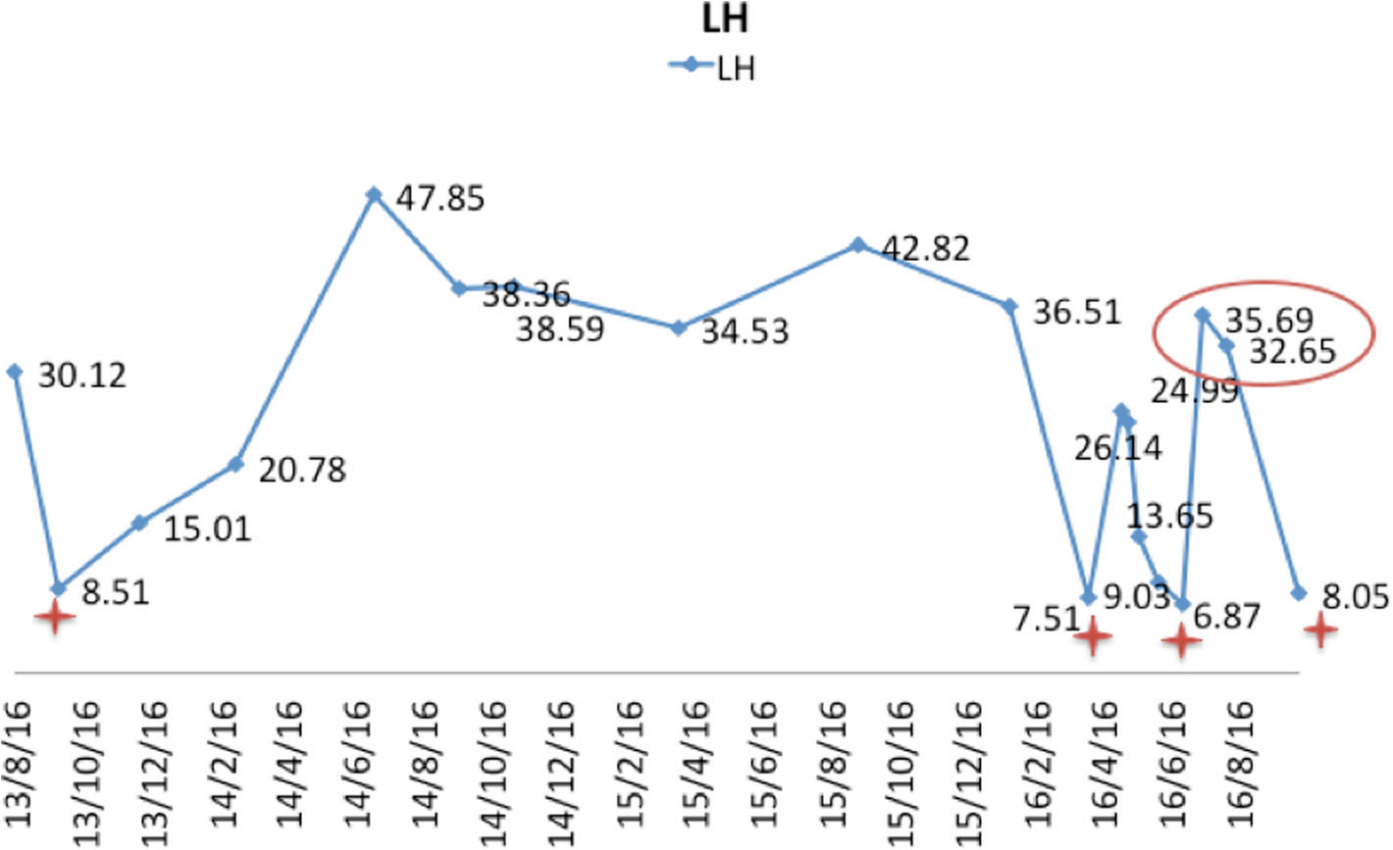
LH level fluctuation during the treatment period.  LH levels exhibited 4 episodes of normalization during the entire treatment course: 2 involving oral contraceptive withdraw bleeding before the surgery, 1 after the left salpingo-oophorectomy and 1 after chemotherapy.  LH levels during the chemotherapy period

Spontaneous menstruation occurred soon after the surgery, and serum hormone levels decreased to normal levels, with LH of 9.03 IU/L and FSH of 8.54 IU/L on the second day of menstruation. The patient received three chemotherapy cycles of cisplatin, etoposide and bleomycin (PEB) as adjuvant treatment. Spontaneous menstruation returned twice during the peri-chemotherapeutic period: one day before and 50 days after chemotherapy. A recent hormonal profile revealed normal LH and FSH levels of 8.05 and 8.27 IU/L. The fluctuations in the LH levels and sex hormone assays during treatment period are summarized in Table 1 and Fig. 2.

**Table 1:** Serum sex hormone assays during the treatment period

		LH IU/L	FSH IU/L	E2 pg/ml	T ng/ml	P ng/ml	PRL ng/ml
	2013/8/16	30.12	7.49	18.2	0.56	0.64	7.12
The second day of menstruation after taking oral contraceptives	2013/9/24	8.51	2.3				
	2013/12/6	15.01	2.59	143.43	0.51	3.94	5.6
The second day of spontaneous menstruation	2014/3/1	20.78	2.63	37.86	0.47	1.26	16.08
Dydrogesterone for 3 months	2014/7/3	47.85	8.76	28.17	0.59	0.42	6.78
	2014/9/18	38.36	8.01	22.89	0.71	0.42	7.61
	2014/11/6	38.59	3.31	11	0.7	1.3	10.88
Artificial cycle	2015/4/2	34.53	5.28	34	0.48	0.41	5.58
	2015/9/11	42.82	3.14	28	0.56	0.29	5.05
	2016/1/25	36.51	2.32	19.07	0.68	0.64	5.56
The second day of menstruation after taking oral contraceptives	2016/4/5	7.51	0.87	<5	0.21	0.64	9.02
Two days before the first surgery	2016/5/4	26.14	2.54	19	0.61	0.71	7.18
Three days after the first surgery	2016/5/9	24.99	6.48	26.09	0.15	0.91	27.28
Two weeks after the first surgery	2016/5/20	13.65	4.91	64	0.38	0.38	6.44
Two days after the second surgery (menstruation occurred after the surgery)	2016/6/6	9.03	8.54	30.23	<0.1	0.69	10.77
Two days before the chemotherapy	2016/6/28	6.87	4.84	138	0.24	11.53	11.95
Chemotherapy	2016/7/15	35.69	58.24	23	0.2	0.98	
Chemotherapy	2016/8/5	32.65	52.34	47	<0.1	0.73	8.01
Fifty days after the chemotherapy	2016/10/10	8.05	8.27

There have been several case reports of GCTs with secondary amenorrhea accompanied by alteration in various sex hormones [6–9]. Most of these cases were associated with low baseline FSH levels and high inhibin levels, but the serum levels of LH and estradiol were essentially normal in the majority of the patients. To the best of our knowledge, there have been only four cases reported [8, 10–12] thus far with a hormonal profile similar to our patient.

## Discussion and conclusions

It was challenging to determine the etiology for the secondary amenorrhea and persistent markedly elevated LH with normal FSH and low E2 in this patient. The ovarian mass after 2 years of amenorrhea exhibited unremarkable growth by pelvic ultrasound. The initial assessment ruled out premature ovarian failure and polycystic ovary syndrome and led to the suspicion of a hypothalamic-pituitary cause. However, pituitary microadenomas that secret LH are rare, and LH that was adequately suppressed using oral contraceptives led us to reject this diagnosis. Thus, surgical exploration of the ovary was performed and confirmed the diagnosis of GCT.

The reason for the elevation of LH levels in the present patient remains not clear. The gonadotropin profile prompted a review of some unusual etiologies of amenorrhea associated with high LH, normal FSH and low estradiol. Some hypotheses are suggested based on a literature review.

The production of LH by the ovarian tumor was ruled out by immunohistochemistry. The GCT may have secreted a factor that could stimulate LH secretion from the pituitary glands. One possible candidate factor is gonadotropin-releasing hormone (GnRH)-like substances, which have been detected in the normal granulosa cells of the ovary [13]. The GCT may have maintained GnRH production during tumorigenesis in this patient. Another possibility for the elevation of LH in GCTs is related to the LH receptor mutation, which is a rare cause of amenorrhea associated with LH elevation [6].

It was also worth noting that the ovarian mass had grown gradually during the 2- year period (2 cm to 3 cm) under conditions of elevated LH and that LH decreased significantly after tumor removal. We wonder whether the relationship between the ovarian tumor and elevated LH levels may have been reciprocal. These two presentations may have influenced and affected by each other. On the one hand, the tumor may have secreted some substance that leads to the increased LH indirectly. Furthermore, elevated LH may have driven the growth of the tumor.

Low estradiol in this patient was unexpected because estradiol is typical one of the first markers identified in patients with GCTs. However, approximately 30% of GCTs do not produce estradiol, perhaps related to the lack of theca cells, which produce androstenedione, a necessary precursor for estradiol synthesis.

Many of our hypotheses are unable to be tested because of ethical or technical restrictions. However, the case we present may provide some clinical basis to doctors who encounter similar cases. Further studies analyzing findings in GCTs associated with elevated serum LH may be helpful to elucidate the mechanisms of LH secretion regulated by the hypothalamus-pituitary-ovary axis.

Although GCTs can typically be diagnosed in the early stages in most patients secondary to the typical hormonal changes, the chance of recurrence remains high. Childbirth should be encouraged for infertile patients following the decline in the LH level and the recovery of normal ovulation after conservative surgery. Most cases in the literature succeeded in achieving natural pregnancy after tumor removal. For patients who failed to achieve natural pregnancy, conception guidance should be recommended.

In addition, monitoring serum LH is necessary and may provide additional tumor markers for such cases after conservative surgery. Patients should also pay attention to the regularity of their cycles, which may be the easiest way to discover possible recurrence. If a patient has an ovarian mass accompanied by endocrine disorders (especially rich blood flow signals on ultrasonography), an operation may be the appropriate diagnostic method, even if the mass is less than 5 cm.

The mechanisms underlying elevated serum LH levels in GCTs require further investigations. However, addressing the clinical problems with respect to the patient’s needs remains the most important current treatment approach.

## Abbreviations

FSH: Follicle stimulating hormone
GCT: Ovarian granulosa cell tumors
GnRH: Gonadotropin-releasing hormone
hCG: Human chorionic gonadotropin
IRB: Institutional Review Board
LH: Luteinizing hormone
MRI: Magnetic resonance imaging
PEB: Chemotherapy of cisplatin, etoposide and bleomycin
